# A Case of Contralateral Left Main Bronchial–Esophageal Fistula Following Right Lower Lobectomy with Systematic Mediastinal Lymph Node Dissection

**DOI:** 10.70352/scrj.cr.26-0082

**Published:** 2026-04-09

**Authors:** Toshiki Tanigawa, Hiroki Ebana, Daisuke Shimizu, Ichiba Hiroki, Ryuhei Sakata, Aki Kobayashi

**Affiliations:** Department of Thoracic Surgery, Tokyo Metropolitan Bokutoh Hospital, Tokyo, Japan

**Keywords:** bronchial–esophageal fistula, pulmonary resection, mediastinal lymph node dissection, soft coagulation, delayed thermal injury

## Abstract

**INTRODUCTION:**

Bronchial or tracheobronchial esophageal fistula following pulmonary resection is a rare complication, and involvement of the contralateral main bronchus is extremely uncommon.

**CASE PRESENTATION:**

A 78-year-old woman underwent right lower lobectomy with systematic mediastinal lymph node dissection for primary lung cancer. Her initial postoperative course was uneventful, and she was discharged on POD 2. On POD 14, however, she presented with coughing and vomiting during oral intake. Further evaluation revealed a bronchoesophageal fistula (BEF) between the contralateral left main bronchus and the esophagus. Review of the intraoperative video did not demonstrate intentional direct manipulation of the left main bronchus or the esophagus; however, inadvertent contact during hemostasis of subcarinal #7 lymph node (LN #7) tissue cannot be completely excluded. Therefore, delayed thermal injury associated with soft coagulation during LN #7 dissection was considered a possible contributing mechanism in this case. The patient was initially managed conservatively with fasting, total parenteral nutrition, and enteral feeding via a jejunostomy, resulting in gradual reduction of the fistula. After adequate resolution of local inflammation, additional endoscopic clipping from the esophageal side was performed, achieving complete closure.

**CONCLUSIONS:**

This case suggests that energy devices, particularly soft coagulation, may be associated with delayed thermal injury even in areas not directly manipulated. Furthermore, a stepwise treatment strategy based on initial conservative management may be effective for small BEFs with localized inflammation in selected cases.

## Abbreviations

BEF
: bronchoesophageal fistula
FEV1
: forced expiratory volume in 1 second
HbA1c
: hemoglobin A1c
LN #7
: subcarinal #7 lymph node

## INTRODUCTION

Tracheoesophageal and BEFs following pulmonary resection are rare complications; however, once established, they represent a life-threatening condition that can lead to aspiration pneumonia, empyema, sepsis, and potentially fatal outcomes. Although sporadic cases of tracheal or BEFs after lung resection have been reported, cases involving fistula formation in the contralateral main bronchus are exceedingly rare. Consequently, the underlying mechanisms and optimal treatment strategies for this condition remain poorly defined.

Herein, we report an extremely rare case of a BEF between the contralateral left main bronchus and the esophagus following right lower lobectomy with systematic mediastinal lymph node dissection. The fistula was successfully treated with a stepwise conservative treatment strategy. This case is clinically noteworthy in that delayed thermal injury associated with the use of energy devices was considered a possible contributing mechanism, and a stepwise treatment strategy centered on conservative management proved effective. We present this case with a review of the relevant literature.

## CASE PRESENTATION

A 78-year-old woman was referred for surgical treatment of suspected primary lung cancer. Her medical history included hypertension and diabetes mellitus (HbA1c 7.5%). Pulmonary function was acceptable for lobectomy (vital capacity 77.9% predicted; FEV1 70.7%).

**Fig. 1:**
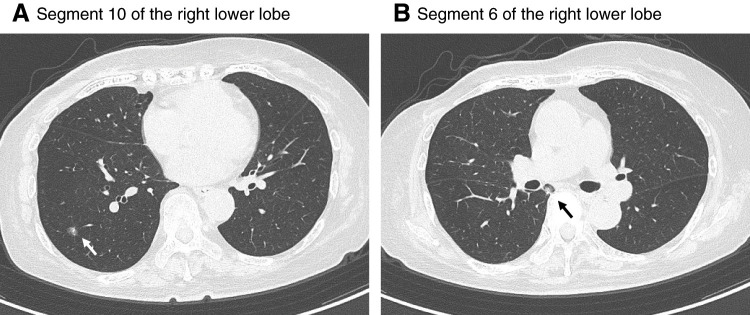
Preoperative chest CT images. (**A**) A slowly enlarging part-solid ground-glass opacity was identified in segment 10 of the right lower lobe, measuring 7.3 mm in total diameter with a 1-mm solid component. (**B**) A newly appeared solid nodule measuring approximately 10 mm was observed in segment 6 of the right lower lobe (arrow).

Chest CT demonstrated a part-solid ground-glass opacity in segment 10 of the right lower lobe, measuring 7.3 mm in total diameter with a 1-mm solid component, which had gradually increased in size compared with findings obtained 8 months earlier (**Fig. 1A**). Another solid nodule measuring approximately 10 mm was observed in segment 6 of the right lower lobe (**Fig. 1B**). No mediastinal lymphadenopathy or pleural effusion was detected.

Based on these findings, the patient was diagnosed with suspected right lower lobe lung cancer (cT1aN0M0, cStage IA1), and surgical resection was planned. Because 2 suspicious lesions were present within the same lobe and intrapulmonary metastasis could not be excluded preoperatively, right lower lobectomy with systematic mediastinal lymph node dissection was selected to ensure oncologic completeness. After discussion of surgical options, the patient and her family preferred definitive resection to minimize the risk of recurrence.

**Fig. 2:**
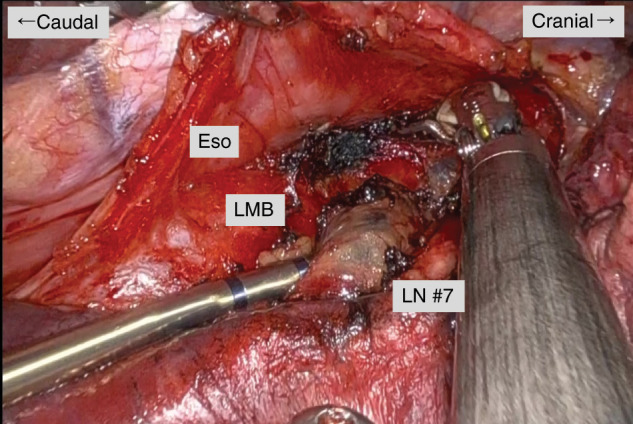
Intraoperative findings. Minor bleeding from LN #7 tissue adjacent to the LBM was controlled using soft coagulation with a ball-type electrode. A darkened area is visible in the coagulated field, which may represent carbonized lymphatic tissue or coagulated blood within the operative field. Cranial and caudal orientation markers are shown. Eso, esophagus; LMB, left main bronchus; LN #7, subcarinal #7 lymph node

Under general anesthesia with one-lung ventilation, the patient was placed in the left lateral decubitus position. Dual-portal robotic-assisted thoracoscopic surgery was performed using a 4-cm incision at the sixth intercostal space along the anterior-to-middle axillary line and an additional 1.8-cm incision at the ventral eighth intercostal space. Right lower lobectomy with systematic mediastinal lymph node dissection was completed without major intraoperative complications. The bronchial stump was covered with pericardial fat tissue. During LN #7 dissection, minor bleeding was encountered and controlled using soft coagulation with a ball-type electrode (**Fig. 2**).

Histopathological examination revealed adenocarcinoma in situ, non-mucinous type, in the segment 10 lesion, corresponding to pTisN0M0, pStage 0. The segment 6 lesion consisted of collapsed and fibrotic tissue without malignant findings.

The postoperative course was initially uneventful. The chest drain was removed on the day of surgery, and the patient was discharged on POD 2.

**Fig. 3:**
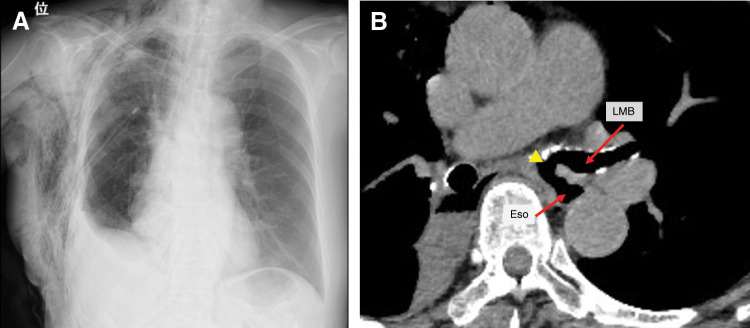
Chest radiograph and CT images at readmission. (**A**) Chest radiography revealed mild collapse of the right lung and subcutaneous emphysema. (**B**) Axial contrast-enhanced CT image at the subcarinal level demonstrating a suspected communication (yellow arrowhead) between the LMB and the Eso. The LMB and the Eso are indicated by red arrows. Eso, esophagus; LMB, left main bronchus

On POD 14, she presented to the emergency department with coughing and vomiting during oral intake. Chest radiography showed mild pneumothorax and subcutaneous emphysema on the operative side, and a chest drain was inserted (**Fig. 3A**). No air leakage was observed, and lung re-expansion was satisfactory; however, persistent coughing prompted further evaluation. Chest CT suggested a communication between the left main bronchus and the esophagus (**Fig. 3B**).

**Fig. 4:**
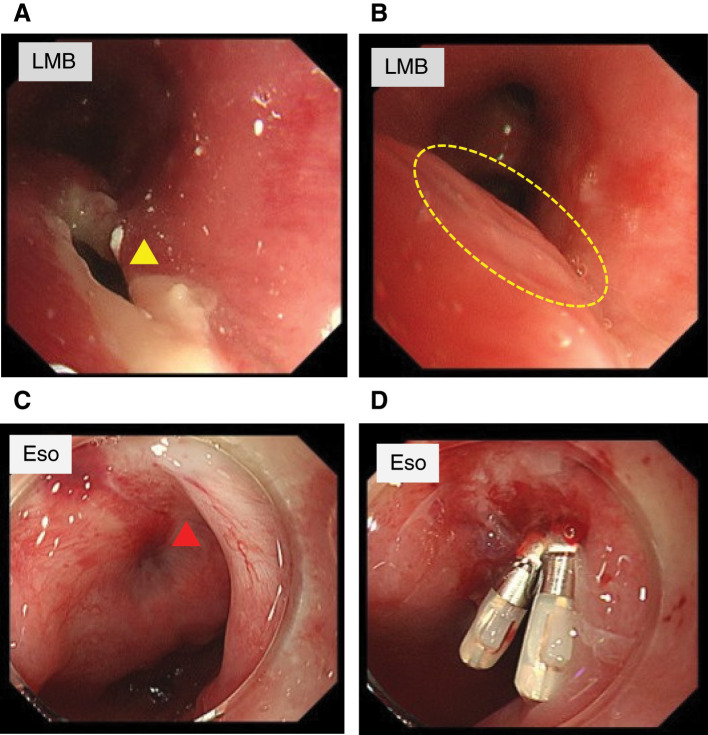
Bronchoscopic and upper gastrointestinal endoscopic findings. (**A**) Bronchoscopic image showing an approximately 1-cm fistula (yellow arrowhead) at the 5 o’clock position on the membranous portion of the LMB. (**B**) Follow-up bronchoscopy performed approximately 2 weeks after diagnosis demonstrating marked reduction of the fistula. The dotted circle indicates the previous fistula site, which appeared as a healing scar with resolution of surrounding inflammation. (**C**) Upper gastrointestinal endoscopic view showing the corresponding fistula opening (red arrowhead) on the Eso side. (**D**) Endoscopic clipping performed from the Eso side to achieve closure. Eso, esophagus; LMB, left main bronchus

Bronchoscopy revealed an approximately 1-cm fistula located at the 5 o’clock position of the membranous portion of the left main bronchus, leading to the diagnosis of a BEF (**Fig. 4A**). Conservative management was selected, including fasting and total parenteral nutrition. A jejunostomy was additionally created to allow enteral feeding. The fistula gradually decreased in size during follow-up. Follow-up bronchoscopy performed approximately 2 weeks after diagnosis demonstrated a marked reduction of the fistula, and the previous fistula site appeared as a healing scar with resolution of surrounding inflammation (**Fig. 4B**).

The chest tube was removed on hospital day 16 after readmission following confirmation of sustained lung expansion without recurrence of pneumothorax. Early mobilization was subsequently encouraged.

On hospital day 33 after readmission, upper gastrointestinal endoscopy demonstrated scar formation at the fistula site (**Fig. 4C**), and endoscopic clipping was performed from the esophageal side (**Fig. 4D**).

**Fig. 5:**
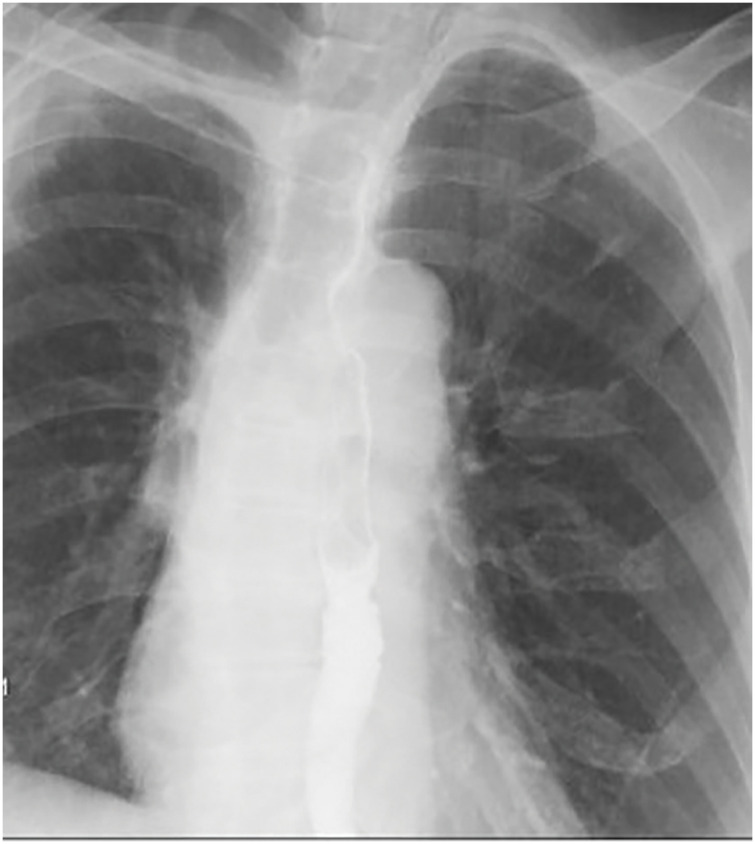
Esophagographic examination. An esophagographic study performed 40 days after readmission showed no leakage of contrast medium into the LMB. LMB, left main bronchus

An esophagographic examination performed on hospital day 40 confirmed the absence of contrast leakage into the left main bronchus (**Fig. 5**), and oral intake was resumed on the same day. The patient was discharged on hospital day 43 without further complications. At the 2-year follow-up, she remained well with no evidence of lung cancer recurrence.

## DISCUSSION

This case represents an extremely rare complication of BEF between the contralateral left main bronchus and the esophagus following right lower lobectomy with systematic mediastinal lymph node dissection. Although tracheal or BEFs after pulmonary resection are uncommon, once established they may lead to serious and potentially fatal complications, including aspiration pneumonia, empyema, and sepsis. In particular, reports describing fistula formation in the contralateral main bronchus are exceedingly limited, making investigation of the underlying mechanisms and optimal management strategies clinically meaningful.

Koike et al. reported a similar case of BEF involving the contralateral main bronchus after lobectomy with systematic mediastinal lymph node dissection, in which the patient was readmitted on POD 13 with coughing after oral intake and dysphagia, and was successfully treated with conservative management.^1)^ While the present case shares similarities with their report, including right lower lobectomy and LN #7 dissection followed by contralateral bronchial involvement, there are notable differences. In our case, soft coagulation using a ball-type electrode was applied as an energy device, and the patient had diabetes mellitus as a systemic comorbidity, both of which may have influenced the clinical course.

The exact mechanism of fistula formation in the present case cannot be definitively determined. There was no evidence of mediastinal infection, anastomotic leakage, or tumor invasion, making these etiologies less likely. Conversely, delayed thermal injury associated with soft coagulation applied during subcarinal lymph node dissection may have contributed to fistula formation. The subcarinal lymph nodes are anatomically adjacent to the tracheal bifurcation, bilateral main bronchi, and the esophagus, and systematic dissection in this region can compromise the surrounding microvasculature, predisposing tissues to ischemic changes. Although soft coagulation is generally regarded as a relatively safe hemostatic modality that avoids surface carbonization, previous reports have indicated that thermal energy may unpredictably propagate to deeper tissues, resulting in delayed necrosis or perforation. In addition, the presence of minor bleeding at the coagulation site may have facilitated local thermal conduction, potentially increasing the risk of unintended heat transfer to adjacent bronchial or esophageal tissue.

Fukunaga et al. reported a case of delayed esophageal perforation occurring on POD 8 after hemostasis using soft coagulation during thoracoscopic right lower lobectomy, demonstrating that severe complications may manifest with a time delay even in the absence of immediately recognizable intraoperative injury.^2)^ A notable feature of the present case is that a BEF developed despite the absence of direct soft coagulation applied to either the esophagus or the contralateral main bronchus. Review of the intraoperative video did not demonstrate intentional direct coagulation or manipulation of the bronchial or esophageal wall. However, the possibility of inadvertent contact between the ball-type electrode and the left main bronchus during hemostasis of LN #7 tissue cannot be completely excluded. It is therefore conceivable that a combination of devascularization caused by subcarinal lymph node dissection and thermal injury associated with soft coagulation gradually led to progressive ischemic damage of the bronchial and esophageal walls, ultimately resulting in delayed fistula formation approximately 2 weeks postoperatively. Rather than a single causative factor, it is more plausible that a combination of devascularization caused by subcarinal lymph node dissection and delayed thermal spread associated with soft coagulation contributed to fistula formation in this case.

Soft coagulation was applied using a VIO3 electrosurgical unit (SOFT COAG mode, effect 8; ERBE Elektromedizin, Tübingen, Germany) with a ball-type electrode for hemostasis of minor bleeding from lymphatic tissue in the subcarinal region. In **Fig. 2**, the coagulated area appears close to the left main bronchus. Although the coagulation was applied to lymphatic tissue in the subcarinal region, minimal unintended contact with the bronchial wall may have occurred during control of minor bleeding. Such contact, even if brief, could have contributed to localized thermal injury. The occurrence of a contralateral lesion following right-sided surgery further suggests that thermal energy may spread more extensively than anticipated through anatomical continuity in the subcarinal region. The contralateral location itself does not prove thermal spread; however, it makes direct mechanical or thermal injury unlikely, thereby supporting the possibility of indirect mechanisms such as ischemia and delayed thermal effects. Shibano et al. reported that tissues with low water content, such as the trachea and bronchi, are particularly susceptible to deep thermal injury from coagulation devices, a finding that is consistent with the presumed mechanism in the present case.^3)^ In addition, the use of a ball-type electrode, which may increase the contact surface area depending on handling, could have facilitated greater diffusion of thermal energy, especially if applied for a prolonged duration at a single site. In the present case, the dual-port robotic approach did not result in restricted visualization or compromised maneuverability in the subcarinal region, and the use of soft coagulation reflected routine hemostatic practice rather than a technical limitation of the surgical approach.

Systemic factors may also have contributed to fistula formation. In the present case, preoperative glycemic control was suboptimal, with an HbA1c level of 7.5%. Diabetes mellitus is known to impair microcirculation and delay wound healing, and has been identified as a risk factor for bronchial stump fistula and esophageal injury. A meta-analysis by Li et al. demonstrated a significantly increased risk of bronchial stump fistula in patients with diabetes undergoing pulmonary resection,^4)^ and Cardinale et al. similarly identified diabetes as an important background factor in addition to devascularization and infection associated with mediastinal lymph node dissection.^5)^ It is therefore plausible that local factors, including devascularization and thermal injury, and systemic factors, such as diabetes mellitus, acted synergistically in the development of the fistula in this case.

Regarding management, we selected a conservative treatment strategy without surgical reconstruction or bronchial stenting, consisting of fasting, strict glycemic control, thoracic drainage, total parenteral nutrition, and enteral feeding via jejunostomy. This approach resulted in gradual reduction of the fistula. After confirmation of scar formation on the esophageal side, the gastroenterology team considered that a minor residual leak could not be completely excluded and therefore applied endoscopic clips to approximate the mucosa and reinforce closure, rather than as a primary intervention during the acute inflammatory phase. Complete closure was subsequently achieved.

Previous reports have similarly demonstrated favorable outcomes with endoscopic treatment combined with appropriate nutritional support for small fistulas,^1)^ and the clinical course in the present case is consistent with these findings. However, surgical reconstruction may be required in cases with large fistulas or uncontrolled intrathoracic infection, underscoring the importance of individualized treatment selection based on the underlying pathology.^6)^

In summary, this case suggests that the use of energy devices, including soft coagulation, may carry a risk of delayed and even contralateral airway or esophageal injury, even in the absence of direct manipulation. Although these observations are hypothesis-generating and do not establish a causal relationship, they underscore the importance of heightened awareness of the potential risks associated with energy device use during mediastinal lymph node dissection. In particular, careful handling of energy devices and meticulous perioperative management may be warranted in patients with diabetes mellitus undergoing lung resection with systematic mediastinal lymph node dissection.

## CONCLUSIONS

We experienced an extremely rare case of BEF between the contralateral left main bronchus and the esophagus following right lower lobectomy with systematic mediastinal lymph node dissection. This case suggests that the use of energy devices, including soft coagulation, may be associated with delayed thermal injury even in areas not directly manipulated, underscoring the importance of careful handling during mediastinal lymph node dissection. In addition, for small BEFs with localized inflammation, a stepwise treatment strategy based on conservative management may represent an effective therapeutic option.

## DECLARATIONS

### Availability of data and materials

All data generated or analyzed during this study are included in this published article.
